# Digital transformation and green total factor productivity: A contingency perspective on spatial and social embeddedness

**DOI:** 10.1016/j.isci.2026.117025

**Published:** 2026-07-31

**Authors:** Yu Chen, Shuangshuang Liu, Qian Zhou

**Affiliations:** 1 School of Economics and Management, Zhengzhou University of Light Industry, ZhengZhou 450000, China; 2 School of Economics and Management, Northwest University, Xi’an 710127, China; 3 Research Institute on Economy and Development of Western China, Northwest University, Xi’an 710127, China; 4 School of Economics, Anhui University, HeFei 230601, China

**Keywords:** digital transformation, DT, green total factor productivity, GTFP, manufacturing firms, market competitiveness, public environmental concern

## Abstract

Green high-quality development requires balancing environmental sustainability with manufacturing growth. Using data from Chinese publicly listed firms from 2012 to 2021, this study demonstrates that digital transformation (DT) significantly increases green total factor productivity (GTFP) in the manufacturing sector. This finding remains robust across endogeneity and specification tests, including instrumental variable approaches and Heckman selection models. Mechanism analysis reveals that both market competition and public environmental concern positively moderate the DT-GTFP nexus. Heterogeneity analysis indicates that this positive impact is more pronounced in the eastern regions, during the early stages of DT, and among small-scale enterprises and non-heavily polluting industries. This study highlights the critical role of manufacturing DT in driving green development and offers flexible policy implications for aligning industrial upgrading with public aspirations for a higher quality of life.

## Highlights

• Manufacturing digital transformation significantly boosts green total factor productivity
• The empirical nexus remains highly robust after adjusting for endogeneity issues
• Market competition and public environmental concern positively moderate the impact
• The positive effect is more pronounced in eastern regions and small-scale firms

## Graphical abstract

**Figure:**
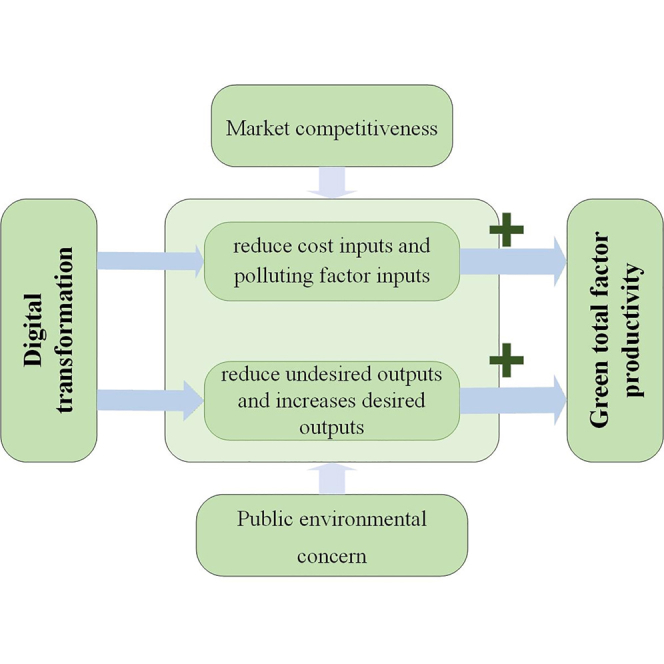

## Teaser

Applied sciences; Manufacturing engineering

## Introduction

Manufacturing is the primary driving force behind China’s economic development, and the green total factor productivity (GTFP) in manufacturing serves as a foundation and prerequisite for achieving high-quality economic growth. It emphasizes the efficient utilization of green resources and fosters sustainable development in the manufacturing sector. GTFP plays a crucial role in advancing industrial upgrading and improving environmental performance.^1^ However, China’s manufacturing sector is still characterized by a relatively high concentration of energy-intensive and pollution-intensive production structures. In terms of energy use, the rapid expansion of manufacturing has contributed to substantial economic growth, but has also been accompanied by a continuous increase in energy consumption. This shift has resulted in serious environmental pollution, particularly regarding industrial sulfur dioxide and particulate matter emissions, leading to ecological degradation. The share of pollution-intensive heavy industries remains relatively high, while emerging green industries account for a comparatively small proportion. For example, in Guangdong Province in 2022, the industrial sector accounted for 58.46% of total national energy consumption, with manufacturing representing the dominant share. Within the sector, energy-intensive industries such as chemicals and steel continue to play a leading role. Of this, the manufacturing sector consumed 1.83 billion tons of standard coal equivalent, accounting for 85% of total industrial energy consumption. Meanwhile, highly polluting and energy-intensive industries such as chemicals and steel continue to dominate the manufacturing sector. Nationally, conventional manufacturing still constitutes a significant portion of the sector’s total value added, while the scale and share of green emerging industries—such as energy conservation, new energy vehicles, and environmental protection—remain underdeveloped. In response to these challenges, building a manufacturing powerhouse requires a strong commitment to sustainable development, with a comprehensive push toward green manufacturing to mitigate conventional industrial pollution and transform production methods that are detrimental to the ecological environment.

In recent years, digital transformation (DT), driven by big data (BD), the internet of things (IoT), and artificial intelligence (AI), has increasingly been regarded as a crucial driver of high-quality development.^2^ DT is the term used to describe how digital technologies are used to fundamentally change business models, processes, decision-making, and value generation within an industry, business, or organization.^3^^,^^4^^,^^5^ China had established 421 nationally designated model plants, alongside more than 10,000 provincially recognized digital workshops and intelligent factories, highlighting the growing role of intelligent manufacturing in advancing industrial modernization and DT. Due to the characteristics of virtuality, inclusiveness, and openness of DT,^6^^,^^7^ digital technologies have the potential to alleviate constraints such as resource scarcity, high investment costs, and technological barriers, thereby creating new opportunities for manufacturing GTFP. For instance, BD technologies enable firms to analyze customer behavior for precise production planning and personalized services, while AI facilitates predictive decision-making based on data-driven learning mechanisms.^8^^,^^9^^,^^10^ Against this backdrop, examining the impact of DT on GTFP in manufacturing and its boundary conditions holds significant importance. Importantly, because our empirical measures of GTFP rely on localized emissions of SO_2_ and smoke/dust rather than carbon metrics, any broader implications discussed herein regarding CO_2_ mitigation or carbon neutrality remain indirect and speculative.

### Literature review and research hypotheses

#### Literature review

##### Literature review on green development

Green development within the manufacturing sector refers to the coordinated development model of “green water and green mountains” and “golden mountains and silver mountains”, guided by the concept of green advancement. It requires establishing an environmentally friendly image and promoting resource-efficient utilization, integrating green practices throughout all aspects of the production system, and pursuing multiple objectives, including environmental sustainability, carbon reduction, and economic benefits.

Currently, research on green development in the industrial sector mainly focuses on two aspects: evaluation and driving factors. The evaluation of the green development level in the manufacturing sector is mainly divided into measuring GTFP and constructing a multidimensional indicator system to calculate the comprehensive score.^11^^,^^12^^,^^13^ This method, which uses input indicators such as capital and labor as well as output indicators such as economic benefits and pollutant emissions,^14^ is widely recognized by academics and calculates the effectiveness of green development using data envelopment analysis.^15^ When scholars establish an indicator system for green development of the manufacturing industry, they mainly measure dimensions such as intensive utilization of factor resources,^16^ the intensity of output pollution emissions,^17^ final production efficiency,^18^ and overall sustainable development capacity.^19^ The second category explores the factors that drive green development within the manufacturing industry. By reviewing relevant literature, scholars have roughly examined the influencing factors from two aspects: their development and the external environment. Internal factors mainly include industrial technology innovation,^20^ human capital level,^21^ and urban or enterprise development concepts that may promote or suppress green development.^22^ External environmental factors include government support,^23^ policy impact degree,^24^ and market competition size.^25^ However, most literature evaluates the green transformation of the manufacturing sector from a macro standpoint, overlooking the evaluation at the micro-entity level and insufficiently examining the influencing mechanisms.

##### Literature review on DT

DT refers to the use of digital technologies by firms to transform traditional industries in terms of their upstream and downstream chains, realizing the empowerment value of digital and information technology enablement through industrial development.^26^ Related studies have focused on transformation-level metrics and drivers. DT level measurement is mainly divided into macro-regional level perspectives and meso-industry level perspectives. At the macro level, studies mostly adopt either the single indicator method or comprehensive evaluation methods to assess regional digitization levels.^27^ At the meso level, they employ the input-output paradigm to calculate the ratio of total IT inputs, communication, and other information services for each industry to characterize the level of digitization.^28^ At the micro level, studies mainly measure manufacturing enterprises’ digitization levels through case study methods.^29^

Digitization-driven research can likewise be categorized into two areas: the role of manufacturing digitalization in promoting enterprise economic growth and its contribution to noneconomic benefits. In terms of digitization-driven economic benefits, the impact of digitization on the growth of the city’s manufacturing sector has been the primary focus of research, from the aspects of digital technology,^30^ digital innovation capacity, and so forth.^31^ Furthermore, studies regarding how businesses are going digital have mainly focused on the promotion of enterprise economic performance from the standpoint of digital technology, in terms of productivity, financial performance, and innovation performance.^32^ In terms of digitization driving the growth of noneconomic benefits, the main positive impacts are related to the degree of internet use in cities and digital economy-related policies,^33^ which drive manufacturing product upgrades and positive impacts on regional entrepreneurial activity. Enterprise digital economy development and DT mainly promote the growth of noneconomic performance, such as structural optimization, quality management, and improvement in factor allocation efficiency, across different dimensions such as governance level, Environmental, Social, and Governance (ESG), and organizational structure.^34^

##### The effect of DT on green development

The Resource Orchestration Theory emphasizes the acquisition, integration, and utilization of resources to achieve their strategic goals.^35^ According to this theory, during the process of DT, enterprises need to acquire various digital technology resources, such as IoT devices, BD analysis platforms, and AI algorithms.^36^ This corresponds to the resource assembly stage of the Resource Orchestration Theory. Secondly, this theory emphasizes bundling different resources together and coordinating them to create greater value. In the context of DT, combining IoT technology with production equipment can enable smart production and optimize energy structures and enhance environmental performance.^37^ Finally, driven by DT, enterprises can achieve precise production through digital production management systems, thereby minimizing raw material waste and significantly reducing the discharge of industrial pollutants, such as sulfur dioxide and soot.^38^

DT can help manufacturing enterprises improve equipment utilization, improve equipment utilization, optimize production processes, shorten production cycles, and more flexibly meet customers’ individual needs by leveraging a variety of digital technologies in response to the constant changes in consumer demand.^39^^,^^40^ In addition, DT can monitor energy consumption in real time, enable precise production and supply chain management, and utilize blockchain technology to facilitate the recycling, reuse, and tracking management of obsolete products, thereby promoting the circular economy.^41^^,^^42^ Therefore, DT has become the future trend for manufacturing enterprises.

Studies on the relationship between DT and green development in the manufacturing industry. By reviewing relevant publications on the influence of DT on green development, it is concluded that such studies mostly present the following three views. First, DT promotes green and high-quality industrial development. Digital technology, which relies on data elements,^43^ exhibits the following traits: high permeability, integration, and innovation,^44^^,^^45^ which can contribute to the green development of the industry through two paths—reduction of incremental quantity and compression of stock.^46^ Second, DT impedes green development in the industry because digital technologies are dependent on electricity consumption,^47^ thus, such technologies not only have limited effects on reducing energy use, but their “rebound effect” may counteract DT’s potential environmental benefits,^48^ thereby failing to promote high-quality, environmentally friendly growth. Third, the connection between DT and green development is nonlinear. The operation and development of DT in the early stage require significant electricity consumption and energy-intensive infrastructure, which may inhibit green development. However, as the digital infrastructure improves and digital technologies become more deeply integrated with enterprises, DT can then drive enterprises toward green development.^49^^,^^50^^,^^51^

Regarding research on DT and green development mechanisms, from the standpoint of the direct action mechanism, DT enables producers to rely on data and algorithms, directly interfacing with consumers,^52^ using real-time collection of their energy data to improve matching and factor allocation efficiency,^53^ reduce nonessential resource inputs and consumption,^54^ and thereby encouraging green development. From the perspective of the indirect mechanism, DT enhances information dissemination within enterprises, between enterprises and consumers, and between enterprises and competitors through the effective utilization of digital technologies. This reduces the information search cost arising from information asymmetry and ensures stable investment in green innovation by enterprises, thereby driving green, low-carbon transformation.^55^ In addition, spatial spillovers and siphon effects from DT on green, low-carbon development. DT will lower pollutant levels and energy use in the surrounding areas through spillover effects,^56^ or produce a siphon effect that impedes the low-carbon green development of peripheral cities.^57^

While previous studies have examined the impact of DT on firms’ GTFP, most existing research primarily focuses on internal mediating mechanisms, such as technological innovation, knowledge creation, organizational capability upgrading, and resource reconfiguration. These studies mainly emphasize how DT indirectly improves green performance through internal efficiency enhancement. In contrast, relatively limited attention has been paid to the contingent role of external environmental conditions, particularly how market competition and public environmental concern shape the effectiveness of DT in promoting GTFP. Moreover, prior studies rarely investigate the dynamic and heterogeneous characteristics of the DT-GTFP relationship across different temporal stages, regions, industries, and firm scales, resulting in an incomplete understanding of the contextual conditions under which DT generates green productivity gains.

To address these gaps, this study develops a contingency-based theoretical framework that situates the effect of DT on GTFP within both market and social environmental contexts. Specifically, this study contributes to the literature in the following aspects. First, this study extends the analytical perspective from internal mediation mechanisms to external contingent conditions. Unlike prior studies that mainly focus on how DT affects GTFP through internal organizational capabilities, this study emphasizes the moderating roles of market competition and public environmental concern, thereby revealing how external environmental conditions influence the effectiveness of DT in enhancing green productivity. This enriches the theoretical understanding of the DT-GTFP relationship from a contingency perspective. Second, this study constructs a multi-dimensional external environment framework by simultaneously incorporating market-based and social environmental factors into the analysis. Specifically, market competition reflects the competitive pressure and resource allocation environment faced by firms, while public environmental concern represents the social supervision and institutional legitimacy pressures surrounding corporate environmental behavior. Examining these two dimensions jointly provides a more systematic framework for understanding the boundary conditions under which DT promotes GTFP. Third, this study further enriches the literature by incorporating temporal, regional, industrial, and firm-level heterogeneity into the analysis. The results reveal that the green productivity effects of DT vary significantly across different development stages of the digital economy, regions, pollution-intensive industries, and firm sizes. This finding highlights the context-dependent nature of DT’s environmental effects and provides a more nuanced understanding of how DT contributes to green development under heterogeneous economic and institutional conditions.

### Research hypotheses

GTFP reflects the efficiency of economic output under environmental constraints, incorporating both desirable outputs and undesirable environmental impacts. It captures the extent to which firms achieve coordinated improvements in economic performance and environmental performance. Against the backdrop of increasingly stringent environmental regulations and growing pressure to improve environmental performance, identifying the strategic drivers of GTFP has become a central issue in management and environmental economics research.

Drawing on dynamic capability theory, firms achieve sustained competitive advantage by continuously integrating, reconfiguring, and transforming internal and external resources in response to rapidly changing environments. DT is not merely the adoption of information technologies; rather, it represents a fundamental organizational transformation centered on data-driven decision-making, process reengineering, and strategic renewal. Through the deep integration of digital technologies into business operations and organizational structures, firms enhance their ability to sense opportunities, seize resources, and reconfigure capabilities. First, DT strengthens firms’ real-time information processing and environmental sensing capabilities, enabling more accurate monitoring of resource utilization and pollutant emissions. This enhanced transparency improves decision quality and reduces inefficiencies associated with information asymmetry. Second, digital technologies facilitate process optimization and cross-functional coordination, thereby improving resource allocation efficiency and reducing resource waste and pollutant emissions. Third, digital platforms promote knowledge integration and technological upgrading, accelerating the diffusion and implementation of cleaner production practices. From the perspective of dynamic capability theory, DT enhances firms’ capability to reconfigure resources and adapt to environmental constraints, thereby improving productivity performance under environmental constraints. As a result, DT is expected to exert a positive effect on firms’ GTFP. Accordingly, this study proposes:

**H1**: DT positively affects firms’ GTFP in the manufacturing industry.

DT can improve firms’ GTFP by enhancing resource allocation efficiency, optimizing production processes, and strengthening information integration capabilities. However, the effectiveness of DT is not only determined by firms’ internal technological capabilities but is also closely associated with external market conditions, particularly firms’ market competitiveness. Firms with stronger market competitiveness generally possess superior resource integration capabilities, broader market networks, and greater strategic flexibility, which enable them to better leverage digital technologies to improve operational efficiency and environmental management performance.

Specifically, highly competitive firms are more capable of converting digital investments into environmentally oriented innovation outcomes and production optimization advantages. Under intensified market competition, firms are also more motivated to utilize digital technologies to reduce operational costs, improve product quality, and strengthen environmental management in order to maintain or enhance their market position. Moreover, stronger market competitiveness can facilitate firms’ access to external knowledge, technological collaboration, and customer feedback, thereby accelerating the integration of digital technologies into cleaner production processes. In contrast, firms with weak market competitiveness may face resource constraints and insufficient organizational support, limiting the effectiveness of DT in promoting GTFP. Therefore, stronger market competitiveness is likely to amplify the positive impact of DT on GTFP. Based on the above analysis, this study proposes the following hypothesis:

**H2**: Market competitiveness positively moderates the relationship between DT and GTFP. Specifically, the promoting effect of DT on GTFP is stronger for firms with higher market competitiveness.

DT enhances firms’ GTFP by enabling better resource allocation, process optimization, and the adoption of sustainable production practices. However, the effectiveness of these digital initiatives may depend on the broader social context in which firms operate. One important contextual factor is public environmental awareness, which reflects the degree of societal attention and concern for environmental protection.

From the perspective of stakeholder theory, firms are influenced by the expectations of critical stakeholders, including local communities, consumers, and the media. High levels of public environmental concern increase the pressure on firms to adopt environmentally responsible practices, thereby shaping strategic decision-making and investment priorities. Similarly, institutional theory suggests that firms seek legitimacy by aligning their practices with prevailing social norms and societal expectations. In environments where environmental awareness is high, firms are more likely to leverage their digital capabilities to implement environmentally oriented innovation activities and improve environmental performance. Therefore, when public environmental awareness is stronger, the positive effects of DT on GTFP are expected to be reinforced. The alignment between internal digital capabilities and external societal expectations strengthens the firm’s ability and motivation to achieve improvements in environmental performance and productivity under environmental constraints. Based on this theoretical reasoning, the following hypothesis is proposed.

**H3**: Public environmental awareness positively moderates the relationship between DT and firms’ GTFP, such that higher levels of public environmental concern strengthen the positive impact of DT on GTFP.

### Study design and data sources

#### Definition of variables

##### Dependent variable

Therefore, in the manufacturing sector, GTFP is measured using the Data Envelopment Analysis Program-Malmquist (DEAP-Malmquist) index.^21^^,^^58^^,^^59^ This paper employs the Data Envelopment Analysis (DEA) methodology based on the DEAP-Malmquist index and utilizes the DEAP 2.1 software for data processing. The measurement mainly adopts the following indices: the quantity of workers in the companies represents the labor input; fixed assets and management costs represent the capital input, a list of all fixed assets is adopted using the perpetual inventory approach; business revenue represents the desired output. SO_2_ and soot emissions serve as indicators of undesirable output. The adjustment coefficients of the various regional pollution indices (the ratio of the regional share in the adjustment coefficients of regional pollution indicators ratio of regional share of national pollutant emissions/ratio of regional share of national industrial output value) are multiplied by the industry’s output coefficients (percentage of industrial output of the firm to that of region) to estimate the SO_2_ and soot emissions of each enterprise.^60^

##### Explanatory variables

Using Python’s text recognition functionality, we collate annual reports of A-share firms and extract all textual content into a data pool for feature word screening. We construct a digital dictionary comprising five dimensions: “BD technology”, “AI technology”, “blockchain technology”, “cloud computing technology”, and “digital technology application” (Table S3). By counting the phrase frequency of feature words, we calculate total word frequencies to measure the extent of DT (Appendix A.1). Utilizing Jieba’s segmentation function, we analyze all samples from four additional perspectives—intelligent manufacturing, modern information systems, digital technology applications, and internet business models—calculating keyword disclosure quantities that serve as alternative indicators of DT for robustness testing.^61^^,^^62^ Metrics for measuring DT based on text mining may suffer from issues such as disclosure bias, time mismatch, and semantic ambiguity. To mitigate the potential impact of these issues on research findings, this paper constructs a robust alternative metric for DT by calculating the proportion of digital-related intangible assets (such as software, information systems, and digital platform assets) relative to total intangible assets at year-end.

##### Control variables

Asset-liability ratio, firm age, profitability, top ten shareholders’ shareholding, cash flow level, management shareholding, capital intensity, and fixed asset ratio were selected as control variables to control firm-level factors affecting firms’GTFP as much as possible. For the treatment method, see Tables 1 and S1.

**Table 1:** Variable description

Variables type	Variables name	Code	Variables description
Dependent variable	green total factor productivity	GTFP	
Explanatory variables	digital transformation	DT	crawling the total word frequency of enterprise digitization-related terms from five aspects
		DTB	a number of disclosures of terms related to enterprise digitization crawled from four other different perspectives
		DTC	the proportion of digital-related intangible assets relative to total intangible assets
Control variables	age of business	Age	current year minus year of incorporation
	asset-liability ratio	Fin	total liabilities of the enterprise as a percentage of total assets
	profitability	Pro	percentage of year-end total profit to operating income of listed companies
	shareholding percentage of top 10 shareholders	Top10	percentage of the total number of shares held by the top 10 shareholders
	cash flow level	Cash	operating activity net cash flows
	management shareholding	Share	percentage of number of shares possessed by governance to total number of shares
	capital intensity	Ci	percentage of gross the company’s fixed assets to the yearly average quantity of workers
	fixed asset ratio	Fa	fixed assets to total assets
Moderator variables	market competitiveness	MC	level of competition in the manufacturing sector
	public environmental concern	Ser	search Index for “Smog” on Baidu Search Engine

To alleviate potential concerns regarding common-source correlation, it should be noted that the DT measure and GTFP indicators are constructed based on fundamentally different data structures and measurement logics. Specifically, DT is derived from text-mining analyses of digital-related semantic disclosures in annual reports, reflecting firms’ digital strategic orientation and technology adoption narratives. In contrast, GTFP is calculated using the DEAP-Malmquist index based on structured production and environmental indicators, including labor input, capital input, operating revenue, and pollutant emissions. Importantly, undesirable outputs are estimated using industry- and region-level pollution adjustment coefficients rather than firms’ textual disclosures. Therefore, the construction of GTFP does not rely on digital-related textual content contained in annual reports, thereby reducing the possibility of mechanical correlation between the two measures. Table illustrates the description of the variables.

##### Moderating variables

Market competitiveness reflects a firm’s relative market position and its ability to maintain competitive advantages within the industry. Firms with stronger market competitiveness are generally more capable of resource integration, market expansion, and strategic adjustment, thereby enabling them to better leverage digital technologies to improve production efficiency and environmental performance. Following existing studies,^63^^,^^64^ this study uses the Lerner index to measure firm-level market competitiveness. Specifically, the firm-level Lerner index is calculated based on the proportion of a firm’s operating revenue to the total operating revenue of firms within the same industry. A higher Lerner index indicates stronger market competitiveness and greater market power.

Internet searches have gradually become the primary way for the public to learn about events. As China’s largest Chinese-language search engine, Baidu search accurately reflects the public’s main areas of concern. Following existing research,^65^ by using the daily average of Baidu index values for residents’ searches of the keyword “smog” and applying a logarithmic transformation to these values, we construct a public environmental concern index for listed companies (Appendix A.2).

#### Data sources and description

Current DT levels show significant variation. To best represent the overall DT situation in China’s manufacturing sector, we selected representative A-share manufacturing firms as our 2012–2021 study sample, excluding ST, ∗ST, PT, and delisted companies with substantial data deficiencies. This yielded an unbalanced panel dataset comprising 13,489 firm-year observations from 2,259 distinct companies. We winsorized continuous variables to mitigate outlier effects. Enterprise financial data primarily came from the China Stock Market & Accounting Research (CSMAR) database, while basic firm characteristics were mainly obtained from the Wind database. Information on listed companies’ annual reports is sourced from the Juchao information network, while city-level data is sourced from the “City Statistical Yearbook.” Manufacturing industry classification follows the Securities and Futures Commission (SFC) standards. All variables underwent logarithmic transformation to reduce multicollinearity effects. This study conducted a Variance Inflation Factor (VIF) test, and the results are shown in Table 2. All VIF values are below 10, with a mean value of 1.23, indicating that the likelihood of multicollinearity in the subsequent regression analysis is relatively low. Further details on data sources, variable construction, and data processing procedures are provided in Tables S1 and S2. Table 3 illustrates descriptive statistics.

**Table 2:** Correlation analysis

	GTFP	DT	Age	Ci	Fin	Fa	Pro	Top10	Cash	Share
GTFP	1.000	–	–	–	–	–	–	–	–	–
DT	0.172∗∗∗	1.000	–	–	–	–	–	–	–	–
Age	0.019∗∗	−0.008	1.000	–	–	–	–	–	–	–
Ci	−0.313∗∗∗	0.021∗	−0.087∗∗∗	1.000	–	–	–	–	–	–
Fin	0.127∗∗∗	−0.0032	0.357∗∗∗	−0.210∗∗∗	1.000	–	–	–	–	–
Fa	−0.178∗∗∗	−0.261∗∗∗	0.128∗∗∗	−0.139∗∗∗	0.1603∗∗∗	1.000	–	–	–	–
Pro	−0.006	−0.020∗∗	−0.203∗∗∗	0.028∗∗∗	−0.385∗∗∗	−0.147∗∗∗	1.000	–	–	–
Top10	−0.0161∗	−0.072∗∗∗	0.1683∗∗∗	−0.127∗∗∗	0.086∗∗∗	0.115∗∗∗	−0.032∗∗∗	1.000	–	–
Cash	0.0252∗∗∗	0.0815∗∗∗	−0.183∗∗∗	0.042∗∗∗	−0.394∗∗∗	−0.345∗∗∗	0.267∗∗∗	−0.023∗∗∗	1.000	–
Share	0.035∗∗∗	0.063∗∗∗	−0.390∗∗∗	0.031∗∗∗	−0.168∗∗∗	−0.126	0.117∗∗∗	−0.182∗∗∗	0.079∗∗∗	1.000

**Table 3:** Descriptive statistics

	(1)	(2)	(3)	(4)	(5)
	N	Mean	SD	Min	max
GTFP	13,489	0.037	0.050	0.000	1.182
DT	13,489	0.009	0.0180	0.000	0.116
DTB	13,489	0.033	0.053	0.000	1.000
Age	13,489	1.876	0.928	0.000	3.258
Ci	13,489	1.068	0.342	0.396	2.216
Fin	13,489	0.327	0.136	0.060	0.624
Fa	13,489	0.201	0.105	0.020	0.482
Pro	13,489	0.085	0.145	−0.990	0.491
Top10	13,489	0.342	0.126	0.129	0.635
Cash	13,489	0.135	0.092	0.012	0.445
Share	13,489	0.099	0.154	0.000	0.517

## Results

**Figure 1:**
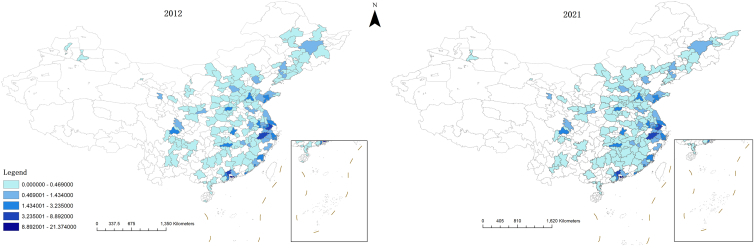
Level of DT in 2012 and 2021

**Figure 2:**
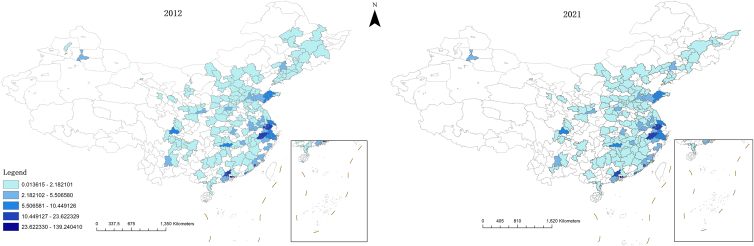
Level of GTFP in 2012 and 2021

To examine the spatiotemporal evolution of manufacturing sector GTFP and DT, we used ArcGIS to visualize GTFP and DT distributions for 2012 and 2021. Figure 1 shows that in 2012, most cities had digitization levels below 0.009, with only certain eastern cities demonstrating higher levels. By 2021, digitization levels showed universal improvement. Figure 2 reveals that in 2011, overall GTFP levels were predominantly below 0.18, while by 2021, GTFP levels had increased substantially.

### Benchmark regression

To address the potential issue of multicollinearity in the multivariate analysis, a stepwise regression strategy was employed, and the results are presented in Tables 4 and S4. In Column (1), which includes firm and year fixed effects but does not control for firm-level characteristics, the coefficient on DT is 0.2230 and statistically significant at the 1% level, indicating a strong positive relationship. In Column (2), after controlling for firm-level characteristics while still including firm and year fixed effects, the DT coefficient remains significantly positive at 0.2030. These findings suggest that DT within firms can effectively enhance their GTFP in manufacturing enterprises. Therefore, Hypothesis 1 is preliminarily supported.

**Table 4:** Baseline regression

	(1)	(2)
	GTFP	GTFP
DT	0.223∗∗∗	0.203∗∗∗
	(0.043)	(0.034)
Constant	0.024∗∗∗	0.073∗∗∗
	(0.002)	(0.005)
Control Variables	–	yes
Company FE	yes	yes
Year FE	yes	yes
R^2^	0.119	0.178
Observations	13,489	13,489

Note: The values in parentheses represent the standard error at the industry level,∗∗∗*p* < 0.01, ∗∗*p* < 0.05, ∗*p* < 0.1.

### Robustness test

#### Replacement of independent variable

To capture DT from different perspectives, this study employs two alternative measures. The primary measure is a text-based DT index derived from annual reports, which reflects firms’ strategic emphasis on digital technologies and the extent to which digitalization is embedded in managerial discourse and organizational narratives. This measure captures firms’ digital orientation and technology adoption intentions, but may be subject to disclosure bias and semantic ambiguity.

First, keywords from the other dimensions are collected. Based on these, a DT keyword dictionary is constructed, and the frequency of each keyword appearing within the respective dimensions is calculated. This frequency is then used to construct an alternative independent variable to evaluate the robustness of the baseline regression results. Second, as a complementary measure, we use the ratio of digital intangible assets to total assets. Unlike the text-based indicator, this measure reflects firms’ actual investments in digital-related assets recorded on the balance sheet and therefore captures the resource commitment dimension of DT. While the text-based measure emphasizes strategic narratives, the intangible-assets measure focuses on realized investments. Consistent findings across these two measures would provide stronger evidence that the results are not driven by a particular operationalization of DT. Specifically, when the detailed items of intangible assets include keywords related to DT technologies—such as “software,” “network,” “client,” “management system,” and “intelligent platform”—as well as related patents, these items are identified as “digital technology intangible assets.” The digital technology intangible assets of the same firm in the same year are then aggregated and divided by the total intangible assets of that year to calculate the proportion, which serves as a proxy variable for the degree of DT (DTC).This measure is used as the explanatory variable to test the robustness of the baseline regression results. As shown in Column (1) and (2) of Tables 5 and S5, this variables continues to exhibit a positive and statistically significant effect on GTFP in manufacturing firms, thereby providing further support for Hypothesis 1.

**Table 5:** Regression results of robustness tests

	(1)	(2)	(3)	(4)	(5)
	GTFP	GTFP	GTFP	GTFP	GTFP
DT	–	–	0.294∗∗∗	0.202∗∗∗	0.203∗∗∗
	–	–	(0.050)	(0.034)	(0.026)
DTB	0.066∗∗∗	–	–	–	–
	(0.014)	–	–	–	–
DTC	–	0.000∗	–	–	–
	–	(0.000)	–	–	–
Control Variables	yes	yes	yes	yes	yes
Company FE	yes	yes	yes	yes	yes
Year FE	yes	yes	yes	yes	yes
Constant	0.072∗∗∗	0.071∗∗∗	0.072∗∗∗	0.074∗∗∗	0.073∗∗∗
	(0.005)	(0.005)	(0.005)	(0.005)	(0.005)
R^2^	0.1770	0.1118	0.1760	0.1770	0.1780
Observations	13,489	13,489	9,855	13,241	13,489

Note: The values in parentheses represent the standard error at the industry level,∗∗∗*p* < 0.01, ∗∗*p* < 0.05, ∗*p* < 0.1.

#### Excluding stock market shocks

Stock liquidity—fundamental to price discovery, information dissemination, resource allocation, and other capital market functions—plays a crucial role in supporting business development. Negative financial events can lead to serious issues such as stock illiquidity,^66^ potentially impeding the DT process. Ignoring such factors may introduce endogeneity concerns. The time-series sample data in this study reveal a major financial shock: the 2015 collapse of the Chinese stock market. This adverse event exhibits lagged effects, prompting the exclusion of firm samples from 2015, 2016, and 2017. As shown in Column (3) of Tables 5 and S5, the DT coefficient in the regression on GTFP remains strongly positive, confirming the robustness of the conclusion.

#### Excluding municipality samples

Given that municipalities often hold special status in terms of legislation, fiscal policy, and governance, the level of DT may vary significantly across regions. To mitigate the potential influence of policy-related and economic structural factors identified in the regression results, a robustness test was conducted by excluding municipal samples. As shown in Column (4) of Tables 5 and S5, the results indicate that the positive impact of DT on GTFP persists, thereby reaffirming the validity of the original conclusion.

#### Change the clustering method

To further ensure that our statistical inferences are not overly sensitive to a specific clustering choice, we conduct an additional robustness check by altering the clustering level of the standard errors. In our baseline specification, standard errors are clustered at the industry level to account for within-industry serial correlation. As a stricter alternative, we re-estimate the baseline models by clustering standard errors at the industry-year level. The results, presented in Column (5) of Tables 5 and S5, demonstrate that the green-enabling effect of DT on corporate GTFP remains statistically significant at the 1% level, reinforcing the empirical reliability of our main findings.

### Endogeneity test

#### Instrumental variables method

Benchmarking and robustness tests help mitigate endogeneity issues stemming from omitted firm and time characteristics, however, concerns such as reverse causality may still persist. To further ensure the reliability of the regression results, IV approach is employed to address problems. Although historical telecommunications infrastructure may be related to current digital development, concerns about exclusion and restrictions remain, as long-standing regional infrastructure conditions may also affect firms’ productivity performance through channels unrelated to DT. Therefore, we use industry-specific annual average DT indicators as instrumental variables. Specifically, the average DT level of other firms in the same industry and year (MDT) is introduced as a instrument. This variable captures industry-level digital trends, which influence a firm’s DT through peer effects (relevance), but do not directly impact the firm’s GTFP (exogeneity). Firms operating within the same industry are exposed to similar technological paradigms, competitive pressures, and digital standards. As digital technologies diffuse across industries, firms tend to imitate successful digital practices adopted by their peers to maintain competitiveness. Therefore, the average level of DT among industry peers is expected to be strongly associated with a focal firm’s DT. The DT activities of other firms in the same industry are unlikely to directly alter the focal firm’s green productivity performance. Instead, their primary influence operates through peer effects that shape the firm’s own DT decisions. Regarding the justification, relevance, and exclusion restriction of MDT, this study conducted rigorous placebo tests. The analysis results are presented in Appendix 4. The results of the analysis are presented in Table S6. As shown in Tables 6 and S7, the regression coefficient on DT remains positive and statistically significant, further confirming the robustness of the findings.

**Table 6:** Regression results of the instrumental variable method

Variables	(1)	(2)
	First	Second
	DT	GTFP
DT	–	1.023∗∗
	–	(0.375)
MDT	0.692∗∗∗	–
	(0.124)	–
Control variables	yes	yes
Company FE	yes	yes
Year FE	yes	yes
K‒P rk LM statistic	4.834∗∗	
K-P rk Wald F statistic	31.134	

Note: The values in parentheses represent the standard error at the industry level,∗∗∗*p* < 0.01, ∗∗*p* < 0.05, ∗*p* < 0.1.

#### Processing effect model

This paper employs Heckman’s two-stage regression to address potential sample selection bias. A DT dummy variable (DT_d) is introduced, assigned a value of 1 if the firm’s DT exceeds the median for the same industry and year, and 0 otherwise. In the first stage, a probit model is constructed with DT_d as the dependent variable to estimate the inverse Mills ratio (IMR). To effectively implement the Heckman two-stage correction and control for potential sample selection bias, we introduce a rigorous exclusion restriction in the first-stage Probit model, we employ the industry-year mean of DT. In the second stage, the IMR is incorporated into the model. The results are presented in Tables 7 and S8. The results of the first stage indicate that the regression coefficient of the exclusionary constraint variable is significantly positive at the 1% significance level. The results of the second stage show that the estimated coefficients of the IMR are all significant, indicating the presence of a certain degree of selection bias; therefore, it is necessary to use the Heckman two-stage selection model for correction. Further comparison reveals that after controlling for sample selection bias, the direction and significance level of the estimated coefficient for DT remain stable, indicating that the results are robust.

**Table 7:** Heckman test results

	(1)	(2)
	First Stage DT_d	Second Stage GTFP
MDT	24.940∗	–
	(15.053)	–
DT	–	0.177∗∗∗
	–	(0.039)
IMR	–	0.013∗∗∗
	–	(0.003)
Control variables	yes	yes
Company FE	yes	yes
Year FE	yes	yes
Observations	13,485	13,254
R-squared/ Pseudo R2	0.654	0.668

Note: The values in parentheses represent the standard error at the industry level,∗∗∗*p* < 0.01, ∗∗*p* < 0.05, ∗*p* < 0.1.

### Moderation effect test

**Figure 3:**
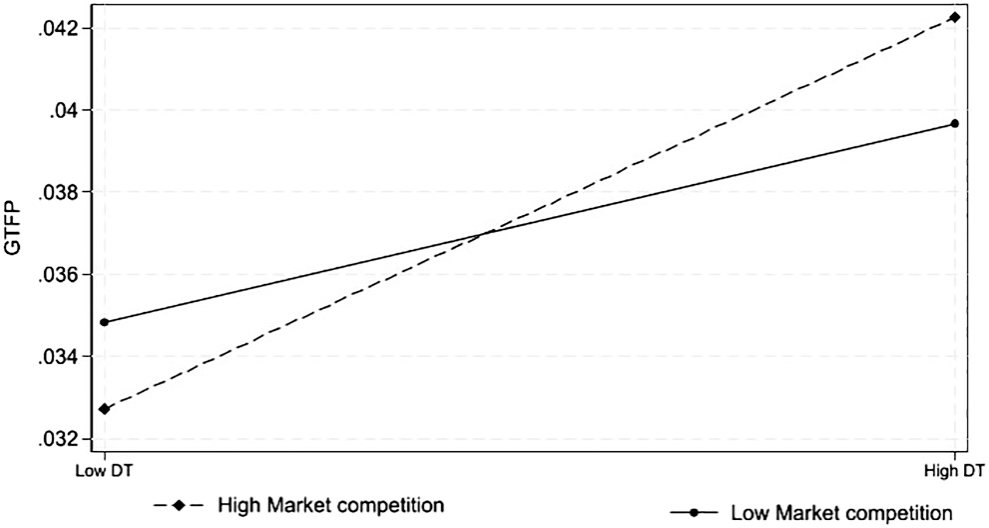
The regulatory role of market competition

As shown in Column (1) of Tables 8 and S9, the interaction terms between market competition and DT are positive, and all interactions passed the 5% significance test, thereby confirming Hypothesis 2. This indicates that the degree of market competition positively moderates the relationship between DT and GTFP. To further elucidate the moderating role of market competitive position in the process by which DT drives corporate innovation, this paper plots a moderation-interaction diagram based on the regression results (Figure 3). The figure illustrates the varying effects of DT on GTFP under different levels of market competition. As can be clearly observed in Figure 2, the slope representing high market competition is significantly greater than that representing low market competition. This indicates that DT has a positive driving effect on GTFP. However, as firms’ market pricing power and competitive position improve, the marginal contribution of DT to GTFP increases significantly. When the level of DT is low, the differences in pollution-related productivity performance among firms with different competitive positions are relatively small. However, as the level of DT rises to higher levels, firms with high market positions exhibit a “leapfrog” lead in GTFP performance. Quantifying this synergistic effect reveals substantial policy relevance. Holding other factors constant, when public environmental concern shifts from a low level (−1 standard deviation) to a high level (+1 standard deviation), the estimated marginal driving effect of DT on corporate GTFP increases from 0.135 to 0.267, representing a net increase of 0.132. This numerical summary suggests that formal technological transformation and informal public supervision are substantively complementary rather than mutually exclusive; informal environmental institutional pressure can nearly double pollution-reduction efficiency gains embedded in firms’ digital investments, making the moderation effect substantively large and highly meaningful for environmental policy targeting.

**Table 8:** Regression results of the moderation effect test

	(1)	(2)
	GTFP	GTFP
DT	0.134∗∗∗	0.163∗∗∗
	(0.028)	(0.038)
MC∗DT	0.474∗∗	–
	(0.191)	–
MC	−0.003	–
	(0.003)	–
PEC∗DT	–	0.117∗∗∗
	–	(0.034)
PEC	–	0.009∗∗∗
	–	(0.003)
Control variables	yes	yes
Company FE	yes	yes
Year FE	yes	yes
Constant	0.073∗∗∗	0.039∗∗∗
	(0.005)	(0.011)
R2	0.178	0.181
Observations	13,489	13,489

Note: The values in parentheses represent the standard error at the industry level,∗∗∗*p* < 0.01, ∗∗*p* < 0.05, ∗*p* < 0.1.

**Figure 4:**
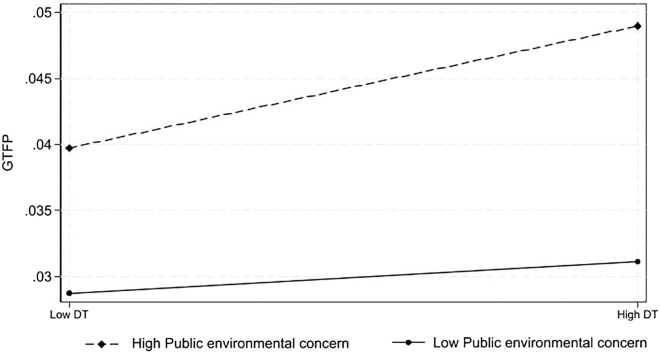
The moderating role of public environmental concern

As shown in column (2) of Tables 8 and S9, the regression coefficient for the interaction term between public environmental concern and DT is significantly positive at the 1% level. This indicates that public environmental concern positively moderates the relationship between DT and GTFP thereby validating Hypothesis 3. To examine the moderating role of public environmental awareness—an external informal institution—on the pollution-constrained productivity effects of DT, this paper constructs an interaction diagram. In Figure 3, the solid and dashed lines represent the effects of DT on GTFP under low and high levels of public environmental awareness, respectively. As shown in Figure 4, the slope of the black dashed line, representing high public environmental awareness, is significantly steeper than that of the black solid line, representing low public environmental awareness. This indicates that as public concern for environmental issues increases, the positive impact of DT on GTFP is significantly amplified. In a high-concern environment, even when DT is at a lower level, the baseline GTFP is significantly higher than in a low-concern environment. As the level of DT advances toward a higher level, the gap between the two widens further, demonstrating a synergistic effect between public pressure and technological transformation. Quantifying this informal institutional channel reveals substantial economic magnitude and critical policy relevance. Holding other control variables constant, when public environmental concern (measured by the Baidu index) is at a low level (−1 standard deviation), the marginal effect of DT on corporate GTFP is small and statistically insignificant (*β* = 0.067, *p* = 0.246). However, when public environmental concern rises to a high level (+1 standard deviation), the marginal pollution-constrained productivity effect of DT is substantially activated, increasing to 0.259 and becoming highly significant at the 1% level (*p* = 0.000). This transition from statistically undetectable to highly significant underscores a vital threshold effect: corporate technological upgrading requires the complementary pressure of external informal institutions to effectively translate into measurable improvements in air-pollution-adjusted productivity.

### Analysis of heterogeneity

#### Regional heterogeneity

Given the substantial regional disparities in digital infrastructure, institutional environment, and innovation capacity in China, the effect of DT on GTFP may vary significantly across different regions. To examine this issue, the sample is divided into eastern, central, and western regions according to firms’ geographic location. The empirical results reveal a pronounced regional disparity in the Tables 9 and S10: the coefficient for the Eastern region is 0.213,significant at the 1% level, whereas the coefficients for the central (0.020) and western (−0.002) regions are statistically insignificant.

**Table 9:** Regional and industry heterogeneity

Variables	(1)	(2)	(3)	(4)	(5)
	Eastern region	Central region	Western region	Highly polluting industries	Non-high-pollution industries
DT	0.213∗∗∗	0.020	−0.002	0.219∗∗∗	0.179∗∗∗
	(0.038)	(0.034)	(0.066)	(0.037)	(0.059)
Control variables	yes	yes	yes	yes	yes
Company FE	yes	yes	yes	yes	yes
Year FE	yes	yes	yes	yes	yes
Constant	0.084∗∗∗	0.044∗∗∗	0.058∗∗∗	0.083∗∗∗	0.058∗∗∗
	(0.005)	(0.008)	(0.013)	(0.007)	(0.003)
R^2^	0.183	0.357	0.248	0.162	0.241
Observations	9,135	2,551	1,803	7,189	5,670

Note: The values in parentheses represent the standard error at the industry level,∗∗∗*p* < 0.01, ∗∗*p* < 0.05, ∗*p* < 0.1.

These findings underscore a significant “digital divide” in the environmentally constrained productivity effect of DT. On the one hand, the Eastern region possesses superior digital infrastructure—such as high-density 5G networks and advanced data centers—which reduces the costs of data transmission and integration for manufacturing firms.^67^ Furthermore, the Eastern region is characterized by a more mature institutional environment and more stringent environmental regulations, compelling firms to utilize digital tools for precise monitoring and abatement of conventional pollutants such as SO_2_ and soot. On the other hand, enterprises in the central and western regions may still face “technological bottlenecks” and limited financial resources. In these areas, the initial costs of digital adoption often outweigh the immediate air-pollution-reduction gains captured by GTFP, resulting in an insignificant impact on GTFP. Consequently, the efficiency gains from DT under air-pollution constraints are currently concentrated in regions with higher levels of marketization and technological readiness.

#### Industry heterogeneity

To further explore the industry-specific impacts of DT on GTFP, we categorize the sample into heavily polluting industries and non-heavily polluting industries based on the 14 types of sectors identified by official environmental guidelines (e.g., thermal power, steel, and cement). As shown in the Tables 9 and S10, the empirical results reveal that the coefficient of DT for heavily polluting industries is 0.219,significant at the 1% level, which is noticeably higher than the 0.179 observed for non-heavily polluting industries. This suggests that the environmentally constrained productivity effect of DT is more pronounced in industries with higher environmental footprints.

Several interwoven mechanisms may account for this finding. First, from a baseline potential perspective, the scope for resource efficiency optimization and conventional pollutant reduction (SO_2_ and soot) is inherently larger in energy-intensive and heavily polluting sectors; thus, integrating digital platforms into these production lines can yield more substantial marginal gains in reducing air-pollution intensity reflected in GTFP. Second, this disparity likely reflects heterogeneous regulatory environments and firm capabilities. Enterprises in heavily polluting sectors are systematically exposed to more stringent environmental regulations, stiffer carbon-reduction targets, and intense administrative oversight, which amplifies their strategic impetus to leverage digital technologies for compliance. Furthermore, heavily polluting industries frequently consist of asset-heavy, well-capitalized firms that possess different technological capabilities and scaling advantages for digital infrastructure compared to their counterparts in cleaner sectors. Therefore, while these results are highly suggestive of a stronger digital-environmental efficiency dividend under air-pollution constraints in high-emission sectors, they should be interpreted as a combined reflection of baseline reduction potential, heightened regulatory pressure, and distinct industry-level capabilities, rather than a definitive single-channel proof. Importantly, since GTFP in this study is constructed based on SO_2_ and soot emissions rather than CO_2_ emissions, any implications for carbon reduction or carbon neutrality are indirect and should be interpreted with caution.

### Temporal heterogeneity

Given the rapid evolution of China’s digital economy and the shifting policy orientation of DT across different development stages, the impact of DT on GTFP may exhibit significant temporal heterogeneity. To explore this issue, this study divides the sample period into an early stage (2012–2015) and a later stage (2016–2021) according to the phased development characteristics of China’s digital economy. The period of 2012–2015 represents the foundational and exploratory stage, during which policy efforts mainly focused on digital infrastructure construction and the diffusion of internet-based business models, such as the “Broadband China” strategy and the “Internet Plus” initiative. In contrast, the period of 2016–2021 reflects the deepening and integration stage, characterized by the transition from consumer-oriented digitalization toward industrial digitalization and the deep integration of digital technologies with the real economy. During this period, policies such as the “National Informatization Development Strategy Outline” and the “Guiding Opinions on Deepening the Integration of Manufacturing and the Internet” further promoted the penetration of digital technologies into production processes and innovation activities.

The results indicate a clear temporal heterogeneity in the effect of DT on GTFP in the Tables 10 and S11. Specifically, in the later stage, the coefficient becomes economically larger and statistically significant at the 1% level. This dramatic shift reflects the “lag effect” and “network effect” inherent in digital technology adoption. During the early stage, high initial investment costs and underdeveloped digital infrastructure limited the capacity of firms to leverage DT for improving pollution-related productivity performance. As digital infrastructure—such as the industrial internet and cloud computing—became pervasive after 2016, the marginal cost of data acquisition dropped significantly. Moreover, manufacturing enterprises have gradually transitioned from simple digitization of office tasks to the deep integration of AI and IoT into core production processes, enabling more precise control over resource consumption and industrial emissions such as SO_2_ and soot. Consequently, the environmentally constrained productivity effect of DT becomes increasingly pronounced as the digital ecosystem matures.

**Table 10:** Heterogeneity across time periods and firm sizes

Variables	(1)	(2)	(3)	(4)
	Early stages	Later stages	Large-scale enterprises	Small-scale enterprises
DT	0.028	0.105∗∗∗	0.247∗∗∗	0.163∗∗∗
	(0.027)	(0.030)	(0.049)	(0.043)
Control variables	yes	yes	yes	yes
Company FE	yes	yes	yes	yes
Year FE	yes	yes	yes	yes
Constant	0.064∗∗∗	0.090∗∗∗	0.064∗∗∗	0.082∗∗∗
	(0.008)	(0.006)	(0.008)	(0.008)
R^2^	0.095	0.130	0.202	0.151
Observations	4,139	9,350	6,745	6,744

Note: The values in parentheses represent the standard error at the industry level,∗∗∗*p* < 0.01, ∗∗*p* < 0.05, ∗*p* < 0.1.

Crucially, this technological transition was highly synchronized with, and catalyzed by, a major regulatory and policy shift in China around 2015–2016, which placed DT within a broader institutional context. On one hand, environmental governance experienced unprecedented tightening during this period, marked by the implementation of the newly revised Environmental Protection Law in 2015 (often dubbed the “strictest in history”) and the official launch of the Central Ecological and Environmental Protection Inspection in 2016. These stringent mandates drastically raised non-compliance risks and environmental costs, transforming DT from an optional technological upgrade into an urgent strategic tool for compliance and pollution control. On the other hand, top-level industrial blueprints issued around this pivot—most notably the “Made in China 2025” strategy and the “National Big Data Strategy” in 2015—explicitly directed the integration of green manufacturing with industrial intelligence. Therefore, the stronger effect of DT in the later stage reflects the joint influence of matured digital infrastructure and intensified environmental regulatory pressure, particularly in relation to conventional air pollutants such as SO_2_ and soot.

### Firm-size heterogeneity

Considering that firm size is closely related to resource endowment, digital capability, and innovation absorption capacity, the impact of DT on GTFP may vary across firms of different scales. In this study, firm size is measured by the number of employees. Firms with values above the sample median are classified as large firms, while those below the median are classified as small firms. As shown in the Tables 10 and S11, the empirical results indicate that the coefficient for large-scale firms is 0.247,significant at the 1% level, which is considerably higher than the 0.163 observed for small-scale firms. While DT promotes GTFP across both groups, the enabling effect is significantly amplified in larger enterprises.

This disparity can be attributed to several factors. First, large-scale firms benefit from economies of scale; the substantial initial capital investment required for digital infrastructure and smart monitoring systems is more easily amortized over a larger volume of output. Second, larger firms typically possess more complex production lines and extensive supply chain data, allowing digital technologies such as BD and AI to achieve more profound optimizations in resource allocation and process control. Finally, large-scale enterprises often face higher social expectations and more rigorous environmental oversight from local regulators.^68^ Consequently, these firms are more motivated to utilize DT to minimize the discharge of conventional pollutants, such as sulfur dioxide and soot, to maintain their corporate reputation and ensure regulatory compliance.

## Discussion

Based on the combined 2012–2021 Wind database, the China Industrial Enterprises Green Development Database, and CSMAR at the manufacturing enterprise level, we explore the relationship between DT and GTFP in manufacturing firms and how such a relationship is shaped by contextual factors. The results show that DT in the manufacturing sector is significantly and positively linked to higher GTFP. This finding is robust across a variety of tests. The level of market competition can amplify the positive association between DT and GTFP. Public environmental concern positively moderates the relationship between DT and GTFP. Heterogeneity analysis indicates that, in the eastern regions and during the early stages of DT, DT can contribute to an increase in GTFP; compared to non-heavily polluting industries and small-scale enterprises, the positive correlation is more prominent. In conclusion, this study demonstrates that DT plays a pivotal role in accelerating enterprise green growth. We explicitly note that while China’s “dual carbon” goals and carbon neutrality commitments represent the ultimate macroscopic policy framework, our empirical evidence evaluates conventional-pollutant-based GTFP. Therefore, our results should be interpreted as a vital piece of the broader ecological upgrading puzzle, rather than a direct metric of CO_2_ reduction. ”

This study offers important theoretical and practical contributions. By adopting a multi-level external environment perspective, the study examines the impact of DT on GTFP, incorporating market competition and public environmental concern as moderating variables, thereby enriching the theoretical understanding of DT and green performance. Unlike prior research that primarily focuses on mediating mechanisms, this study highlights how DT effects may be contingent on spatial and institutional contexts, offering a novel perspective on the green effects of digitalization. By considering regional and firm size differences, the study emphasizes the context-dependent nature of DT’s green effects, enhancing theoretical understanding of multi-level environmental and firm-specific contingencies.

### Theoretical contributions

First, by adopting a multi-level external environment perspective, the study examines the impact of DT on GTFP, incorporating market competitiveness and public environmental concern as moderating variables, thereby enriching the theoretical understanding of DT and environmental performance. Second, unlike prior research that primarily focuses on mediating mechanisms, this study highlights how DT effects may be contingent on spatial and institutional contexts, offering a novel perspective on the environmental effects of digitalization. Third, by considering regional and firm-size heterogeneity, the study emphasizes that the effects of DT on GTFP are context-dependent, thereby enhancing understanding of multi-level institutional and firm-specific determinants of environmentally constrained productivity.

### Practical implications

The empirical findings of this study demonstrate that DT serves as a meaningful facilitator for enhancing GTFP in manufacturing firms, a relationship robustly moderated by market competition, public environmental concern, and regional and firm-level contexts. Although the associational design warrants caution in making strong causal claims, the robustness of the results, supported by IV and Heckman estimations, provides useful insights for policy design. To translate these findings into governance implications, policymakers may consider a two-stage framework distinguishing short-term operational instruments from long-term strategic orientation.

In the short and medium term, policy interventions should leverage digital technologies as targeted tools for local air-pollution management (SO_2_ and soot-related emissions), consistent with the empirical GTFP measure used in this study. Governments may introduce financial subsidies, tax incentives, and technical assistance to encourage firms to adopt digital technologies in production monitoring and environmental management. Priority should be given to intelligent real-time emission monitoring systems and automated end-of-pipe treatment technologies that improve pollution-reduction efficiency at the source. Given the moderating roles of market competition and public environmental concern, policy design should also emphasize improving market transparency and strengthening environmental information disclosure. These measures can enhance competitive pressure and social oversight, thereby increasing firms’ incentives to adopt pollution-control-oriented DT. In addition, consistent with observed heterogeneity, policy support should be differentiated across regions and firm sizes. Eastern regions and large firms may be encouraged to adopt advanced industrial internet platforms for fine-grained emission monitoring, whereas central and western regions, as well as small firms, require stronger financial and technical support to overcome barriers to basic digital and environmental technology adoption.

While the empirical evidence in this study is based on SO_2_- and soot-related GTFP, the observed efficiency gains may generate indirect and long-term spillover effects for broader environmental policy agendas, including China’s “dual carbon” and carbon neutrality goals. However, such implications are speculative and indirect, as the dependent variable does not capture greenhouse gas emissions. Digital-driven improvements in conventional air-pollution control may share partial technological complementarities with energy efficiency enhancements, such as improved process optimization and resource utilization. Therefore, digital monitoring systems developed for air pollutants may serve as a foundational infrastructure for more comprehensive environmental governance systems in the future. Rather than interpreting DT as a direct pathway to decarbonization, the findings should be understood as evidence of improved air-pollution-adjusted production efficiency in manufacturing. Embedding environmental monitoring requirements into ongoing digital economy and smart manufacturing strategies may gradually strengthen the institutional and technological foundation for longer-term environmental transitions.

### Limitations of the study

Despite its theoretical and empirical contributions, this study is subject to several limitations. First, the sample used in this study focuses primarily on publicly listed manufacturing firms in China, which may limit the generalizability and applicability of our empirical findings to a broader context. Second, regarding the analytical mechanisms, this study primarily examines external environmental and market moderating factors, leaving the internal operational mechanisms less explored. Specifically, the macro- and meso-level data utilized herein restrict our ability to capture the micro-level nuances and internal contextual variations within individual manufacturing enterprises.

### Future research directions

These limitations, however, offer valuable and inspiring directions for future research. First, future studies could broaden the sample boundaries to include non-listed manufacturing firms and enterprises in other emerging or developed countries, thereby testing the geographic robustness and institutional boundary conditions of DT’s impact on GTFP. Second, future research could conduct in-depth field studies or case investigations within manufacturing firms to explore potential internal contextual conditions—such as organizational structures, management practices, or technology adoption levels—to further uncover the internal pathways through which DT influences green performance. By integrating a broader sample and more detailed, multi-level mechanism analyses, future research can provide more comprehensive support for both theoretical development and policy practice.

## Resource availability

### Lead contact

Further information and requests should be directed to the lead contact, Dr. Qian Zhou (z0005072@zuel.edu.cn).

### Materials availability

No new materials were generated by this work.

### Data and code availability

This paper analyzes existing, publicly available data which are listed in the key resources table. All data reported in this paper will be shared by the lead contact upon request. Code for the analysis was written in STATA and is available from the lead contact upon request. Any additional information required to reanalyze the data reported in this paper is available from the lead contact upon request.

## Acknowledgments

This work was supported by grants from Support Plan for Scientific and Technological Innovation Talents in Henan Institutions of Higher Learning (humanities and social sciences) (grant no. 2018-cx-012);Good Scholar in Philosophy and Social Sciences in Henan Institutions of Higher Learning (grant no. 2019-YXXZ-20);Philosophy and Social Science Innovation Team Building Program of 10.13039/501100003789Helwan University (grant no. 2021-CXTD-12); Major Project of Applied Research in Philosophy and Social Sciences of Henan Province Higher Education Institutions: Diagnosis of Spatial Conflict and Optimal Regulation of the Yellow River Basin in Three Life Spaces (grant no. 2022-YYZD-27); and Henan Provincial Social Science Planning Project: Study on the Mechanism of Spatial Evolution and Optimal Regulation of the Yellow River Basin in Three Life Spaces (grant no. 2021BJJ112); and Major projects of China Social Science Foundation (grant nos. 18VSJ036, 21ZDA115);Major Project of the Key Research Institute of Humanities and Social Sciences, 10.13039/501100002338Ministry of Education of China (grant no. 22JJD630017); Northwest University Graduate Student Innovation Program for the 2025–2026 Academic Year (grant no. CX2026009); Xi’an Soft Science Project (grant no. 25RKYJ0053).

## Author contributions

Y.C., conceptualization, supervision, writing—review; S.L., methodology, software, writing—original draft preparation, and visualization; Q.Z., supervision and writing—review and editing.

## Declaration of interests

The authors declare no competing interests.

## STAR★Methods

### Key resources table

**Table:** 

REAGENT or RESOURCE	SOURCE	IDENTIFIER
**Deposited data**		

Green Total Factor Productivity	China City Statistical Yearbook; China Stock Market & Accounting Research Database; Wind Database	https://data.cnki.net/; https://data.csmar.com/; https://www.wind.com.cn/
Digital Transformation	Annual Reports of Chinese Listed Companies	https://uc.cninfo.com.cn
Age of business	Wind Database	https://www.wind.com.cn/
Asset-liability ratio	China Stock Market & Accounting Research Database	https://data.csmar.com/
Profitability	China Stock Market & Accounting Research Database; Wind Database	https://data.csmar.com/; https://www.wind.com.cn/
Shareholding percentage of top 10 shareholders	China Stock Market & Accounting Research Database	https://data.csmar.com/
Cash flow level	China Stock Market & Accounting Research Database	https://data.csmar.com/
Management Shareholding	China Stock Market & Accounting Research Database	https://data.csmar.com/
Capital intensity	China Stock Market & Accounting Research Database	https://data.csmar.com/
Fixed asset ratio	China Stock Market & Accounting Research Database	https://data.csmar.com/
Market competitiveness	China Stock Market & Accounting Research Database; Wind Database	https://data.csmar.com/; https://www.wind.com.cn/
Public environmental concern	Baidu Search Engine	https://www.baidu.com/Index.htm

**Software and algorithms**		

ArcGIS	ESRI	https://www.arcgis.com/index.html
Stata software	Stata	https://www.stata.com/

### Method details

#### Benchmark effect model

To test the theories put forth in the preceding section, the model is established as follows.^69^

(Equation 1) $$GTFP_{it}=\alpha_{0}+\alpha_{1}DT_{it}+\alpha_{2}controls+\lambda_{i}+\lambda_{t}+\varepsilon_{it}$$

The dependent variable in Equation 1 is the GTFP, and the enterprise attribute factors and financial indicators are chosen to serve as the model’s control variables, *λ* and *λ*_*t*_ represent the control year and enterprise effects respectively. The random error terms are added. To more accurately depict the estimated coefficients’ real variability and improve the regression findings’ validity, standard errors are clustered at the industry level.

#### Moderation model

Based on our analysis of the moderation mechanisms, we believe that market competition and public environmental concern positively moderate the relationship between DT and GTFP. The test model is constructed as follows.

(Equation 2) $$GTFP_{it}=\beta_{0}+\beta_{1}M_{it}\times DT_{it}+\beta_{2}M_{it}+\beta_{3}DT_{it}+\beta_{4}controls+\lambda_{i}+\lambda_{t}+\varepsilon_{it}$$

Where *M*_*it*_ refers to the mechanism variables, namely MC,PEC in Equation 2.

### Quantification and statistical analysis

Statistical analyses were performed using Stata software (version 18). Significance was assessed using *p*-values, with significance levels indicated by asterisks in tables. ∗*p* < 0.1, ∗∗*p* < 0.05, ∗∗∗*p* < 0.01 (as defined in each table note). Results are considered statis tically significant if *p* < 0.1.

## References

[1] Zheng W., Zhang L., Hu J. (2022). Green credit, carbon emission and high quality development of green economy in China. Energy Rep..

[2] Pan W., Xie T., Wang Z., Ma L. (2022). Digital economy: An innovation driver for total factor productivity. J. Bus. Res..

[3] Ghosh S., Hughes M., Hodgkinson I., Hughes P. (2022). Digital transformation of industrial businesses: A dynamic capability approach. Technovation.

[4] Gillani F., Chatha K.A., Jajja S.S., Cao D., Ma X. (2024). Unpacking Digital Transformation: Identifying key enablers, transition stages and digital archetypes. Technol. Forecast. Soc. Change.

[5] Nadkarni S., Prügl R. (2021). Digital transformation: a review, synthesis and opportunities for future research. Manag. Rev. Q..

[6] Tang M., Liu Y., Hu F., Wu B. (2023). Effect of digital transformation on enterprises' green innovation: Empirical evidence from listed companies in China. Energy Econ..

[7] Luo S., Yimamu N., Li Y., Wu H., Irfan M., Hao Y. (2023). Digitalization and sustainable development: How could digital economy development improve green innovation in China?. Bus. Strat. Environ..

[8] Zheng G., Brintrup A. (2025). Enhancing supply chain visibility with generative AI: an exploratory case study on relationship prediction in knowledge graphs. Int. J. Prod. Res..

[9] Bag S., Pretorius J.H.C., Gupta S., Dwivedi Y.K. (2021). Role of institutional pressures and resources in the adoption of big data analytics powered artificial intelligence, sustainable manufacturing practices and circular economy capabilities. Technol. Forecast. Soc. Change.

[10] Fang W., Guo Y., Liao W., Ramani K., Huang S. (2020). Big data driven jobs remaining time prediction in discrete manufacturing system: a deep learning-based approach. Int. J. Prod. Res..

[11] Li G., Liao F. (2022). Input digitalization and green total factor productivity under the constraint of carbon emissions. J. Clean. Prod..

[12] Qu C., Shao J., Cheng Z. (2020). Can embedding in global value chain drive green growth in China's manufacturing industry?. J. Clean. Prod..

[13] Guo J., Sun Z. (2023). How does manufacturing agglomeration affect high-quality economic development in China?. Econ. Anal. Pol..

[14] Lee C.C., Lee C.C. (2022). How does green finance affect green total factor productivity? Evidence from China. Energy Econ..

[15] Xie R., Yao S., Han F., Zhang Q. (2022). Does misallocation of land resources reduce urban green total factor productivity? An analysis of city-level panel data in China. Land Use Policy.

[16] Zameer H., Wang Y., Vasbieva D.G., Abbas Q. (2021). Exploring a pathway to carbon neutrality via reinforcing environmental performance through green process innovation, environmental orientation and green competitive advantage. J. Environ. Manag..

[17] Alam M.S., Atif M., Chien-Chi C., Soytaş U. (2019). Does corporate R&D investment affect firm environmental performance? Evidence from G-6 countries. Energy Econ..

[18] Baloch M.A., Ozturk I., Bekun F.V., Khan D. (2021). Modeling the dynamic linkage between financial development, energy innovation, and environmental quality: Does globalization matter?. Bus. Strat. Environ..

[19] Ali S., Dogan E., Chen F., Khan Z. (2021). International trade and environmental performance in top ten-emitters countries: The role of eco-innovation and renewable energy consumption. Sustain. Dev..

[20] Chhabra M., Giri A.K., Kumar A. (2022). Do technological innovations and trade openness reduce CO2 emissions? Evidence from selected middle-income countries. Environ. Sci. Pollut. Res. Int..

[21] Wang M., Xu M., Ma S. (2021). The effect of the spatial heterogeneity of human capital structure on regional green total factor productivity. Struct. Change Econ. Dynam..

[22] Carfora A., Scandurra G., Thomas A. (2021). Determinants of environmental innovations supporting small- and medium-sized enterprises sustainable development. Bus. Strat. Environ..

[23] Fang Z., Kong X., Sensoy A., Cui X., Cheng F. (2021). Government’s awareness of Environmental protection and corporate green innovation: A natural experiment from the new environmental protection law in China. Econ. Anal. Pol..

[24] He W., Ding Q., Zhou T. (2025). How fiscal policy drives corporate digital transformation: an analysis of the synergistic effects of tax incentives and special subsidies. Int. Rev. Econ. Finance.

[25] Liu D., Zhu X., Wang Y. (2021). China's agricultural green total factor productivity based on carbon emission: An analysis of evolution trend and influencing factors. J. Clean. Prod..

[26] Verhoef P.C., Broekhuizen T., Bart Y., Bhattacharya A., Qi Dong J., Fabian N., Haenlein M. (2021). Digital transformation: A multidisciplinary reflection and research agenda. J. Bus. Res..

[27] Ren S., Hao Y., Xu L., Wu H., Ba N. (2021). Digitalization and energy: How does internet development affect China's energy consumption?. Energy Econ..

[28] Wen H., Lee C.C., Song Z. (2021). Digitalization and environment: how does ICT affect enterprise environmental performance?. Environ. Sci. Pollut. Res. Int..

[29] Ranta V., Aarikka-Stenroos L., Väisänen J.M. (2021). Digital technologies catalyzing business model innovation for circular economy—Multiple case study. Resour. Conserv. Recycl..

[30] Hao X., Li Y., Ren S., Wu H., Hao Y. (2023). The role of digitalization on green economic growth: Does industrial structure optimization and green innovation matter?. J. Environ. Manag..

[31] Allam Z., Dhunny Z.A. (2019). On big data, artificial intelligence and smart cities. Cities.

[32] Ribeiro-Navarrete S., Botella-Carrubi D., Palacios-Marqués D., Orero-Blat M. (2021). The effect of digitalization on business performance: An applied study of KIBS. J. Bus. Res..

[33] Niu Y., Wen W., Wang S., Li S. (2023). Breaking barriers to innovation: The power of digital transformation. Finance Res. Lett..

[34] Amankwah-Amoah J., Khan Z., Wood G., Knight G. (2021). COVID-19 and digitalization: The great acceleration. J. Bus. Res..

[35] Sirmon D.G., Hitt M.A., Ireland R.D., Gilbert B.A. (2011). Resource orchestration to create competitive advantage: Breadth, depth, and life cycle effects. J. Manag..

[36] Ahn M.J., Chen Y.C. (2022). Digital transformation toward AI-augmented public administration: The perception of government employees and the willingness to use AI in government. Gov. Inf. Q..

[37] Wong C.W.Y., Wong C.Y., Boon-itt S. (2018). How does sustainable development of supply chains make firms lean, green and profitable? A resource orchestration perspective. Bus. Strat. Environ..

[38] Oertwig N., Gering P., Thomas Knothe P.D.I., Rimmelspacher S.O. (2019). User-Centric Process Management System for Digital Transformation of Production. Procedia Manuf..

[39] Wang D., Shao X. (2024). Research on the impact of digital transformation on the production efficiency of manufacturing enterprises: Institution-based analysis of the threshold effect. Int. Rev. Econ. Finance.

[40] Sagar S. (2024). The impact of digital transformation on retail management and consumer behavior. J. Bus. Manag..

[41] Banihashemi S., Meskin S., Sheikhkhoshkar M., Mohandes S.R., Hajirasouli A., LeNguyen K. (2024). Circular economy in construction: The digital transformation perspective. Clean. Eng. Technol..

[42] Okorie O., Russell J., Cherrington R., Fisher O., Charnley F. (2023). Digital transformation and the circular economy: Creating a competitive advantage from the transition towards Net Zero Manufacturing. Resour. Conserv. Recycl..

[43] Dong K., Yang S., Wang J. (2023). How digital economy lead to low-carbon development in China? The case of e-commerce city pilot reform. J. Clean. Prod..

[44] Caragliu A., Del Bo C.F. (2019). Smart innovative cities: The impact of Smart City policies on urban innovation. Technol. Forecast. Soc. Change.

[45] Cheba K., Kiba-Janiak M., Baraniecka A., Kołakowski T. (2021). Impact of external factors on e-commerce market in cities and its implications on environment. Sustain. Cities Soc..

[46] Usman A., Ozturk I., Ullah S., Hassan A. (2021). Does ICT have symmetric or asymmetric effects on CO2 emissions? Evidence from selected Asian economies. Technol. Soc..

[47] Lin B., Huang C. (2023). How will promoting the digital economy affect electricity intensity?. Energy Policy.

[48] Shi X., Geng S. (2025). Double-edged sword? Heterogeneous effects of digital technology on environmental regulation-driven green transformation. J. Environ. Manag..

[49] Zhang L., Mu R., Zhan Y., Yu J., Liu L., Yu Y., Zhang J. (2022). Digital economy, energy efficiency, and carbon emissions: Evidence from provincial panel data in China. Sci. Total Environ..

[50] Amuso V., Poletti G., Montibello D. (2020). The Digital Economy: Opportunities and Challenges. Glob. Policy.

[51] Dwivedi Y.K., Hughes L., Kar A.K., Baabdullah A.M., Grover P., Abbas R., Andreini D., Abumoghli I., Barlette Y., Bunker D. (2022). Climate change and COP26: Are digital technologies and information management part of the problem or the solution? An editorial reflection and call to action. Int. J. Inf. Manag..

[52] Guo Q., Wang Y., Dong X. (2022). Effects of smart city construction on energy saving and CO2 emission reduction: Evidence from China. Appl. Energy.

[53] Chauhan A., Jakhar S.K., Chauhan C. (2021). The interplay of circular economy with industry 4.0 enabled smart city drivers of healthcare waste disposal. J. Clean. Prod..

[54] Nizam H.A., Zaman K., Khan K.B., Batool R., Khurshid M.A., Shoukry A.M., Sharkawy M.A., Aldeek F., Khader J., Gani S. (2020). Achieving environmental sustainability through information technology: “Digital Pakistan” initiative for green development. Environ. Sci. Pollut. Res. Int..

[55] Zhang W., Xu N., Li C., Cui X., Zhang H., Chen W. (2023). Impact of digital input on enterprise green productivity: Micro evidence from the Chinese manufacturing industry. J. Clean. Prod..

[56] Wan Y., Sheng N., Wei X., Su H. (2023). Study on the spatial spillover effect and path mechanism of green finance development on China's energy structure transformation. J. Clean. Prod..

[57] Kunkel S., Tyfield D. (2021). Digitalisation, sustainable industrialisation and digital rebound – Asking the right questions for a strategic research agenda. Energy Res. Social Sci..

[58] Hong Q., Cui L., Hong P. (2022). The impact of carbon emissions trading on energy efficiency: evidence from quasi-experiment in China's carbon emissions trading pilot. Energy Econ..

[59] Meng Y., Yu J., Yu Y., Myšková R. (2024). Impact of green finance on green total factor productivity: New evidence from improved synthetic control methods. J. Environ. Manag..

[60] Zhu Q., Liu C., Li X., Zhou D. (2023). The total factor carbon emission productivity in China’s industrial Sectors: An analysis based on the global Malmquist-Luenberger index. Sustain. Energy Technol. Assessments.

[61] Su Y., Wu J. (2024). Digital transformation and enterprise sustainable development. Finance Res. Lett..

[62] Zhang H., Wu J., Mei Y., Hong X. (2024). Exploring the relationship between digital transformation and green innovation: The mediating role of financing modes. J. Environ. Manag..

[63] Wang Y., Zhao Z., Shi M., Liu J., Tan Z. (2024). Public environmental concern, government environmental regulation and urban carbon emission reduction—Analyzing the regulating role of green finance and industrial agglomeration. Sci. Total Environ..

[64] Qiwen D., Ju H., Zhongyuan G., Yanqiao Z., Yue Z. (2025). Green finance for sustainable development: Analyzing the effects of green credit on high-polluting firms' environmental performance. Hum. Soc. Sci. Commun..

[65] Wang Z., Sun H., Ding C., Zhang X. (2024). Does public environmental concern cause pollution transfer? Evidence from Chinese firms' off-site investments. J. Clean. Prod..

[66] Bongaerts D., Roll R., Rösch D., van Dijk M., Yuferova D. (2022). How do shocks arise and spread across stock markets? A microstructure perspective. Manag. Sci..

[67] Cheng M., Shao Z., Gao F., Yang C., Tong C., Yang J., Zhang W. (2020). The effect of research and development on the energy conservation potential of China’s manufacturing industry: The case of east region. J. Clean. Prod..

[68] von Garrel J., Jahn C. (2023). Design Framework for the Implementation of AI-based (Service) Business Models for Small and Medium-sized Manufacturing Enterprises. J. Knowl. Econ..

[69] Demirci O., Hannane J., Zhu X. (2025). Who Is AI Replacing? The Impact of Generative AI on Online Freelancing Platforms. Manag. Sci..

