# Supplementary material for: Digital transformation and green total factor productivity: A contingency perspective on spatial and social embeddedness
Source: iScience. 2026 Jul 31;29(8):117025. doi: 10.1016/j.isci.2026.117025 (PMC13453038; doi:10.1016/j.isci.2026.117025)
Supplement: Supplementary file 1 [file mmc1.pdf]

iScience, Volume *29*

Supplemental information

Digital transformation and green total factor productivity: A contingency perspective on spatial and social embeddedness

Yu Chen, Shuangshuang Liu, and Qian Zhou

Table S1 Variable description

Variables name	Code	Calculation Method
Green Total Factor Productivity	GTFP	The measurement mainly adopts the following indices: the quantity of workers in the companies represents the labor input; fixed assets and management costs represent the capital input, a list of all fixed assets is adopted using the perpetual inventory approach; business revenue represents the desired output. SO_2 and soot emissions serve as indicators of undesirable output
Digital Transformation	DT	Crawling the total word frequency of enterprise digitization-related terms from five aspects
Age of business	Age	Current year minus year of incorporation
Asset-liability ratio	Fin	Total liabilities of the enterprise as a percentage of total assets
Profitability	Pro	Percentage of year-end total profit to operating income of listed companies
Shareholding percentage of top 10 shareholders	Top10	Percentage of the total number of shares held by the top 10 shareholders
Cash flow level	Cash	Operating activity net cash flows
Management Shareholding	Share	Percentage of number of shares possessed by governance to total number of shares
Capital intensity	Ci	Percentage of gross the company's fixed assets to the yearly average quantity of workers
Fixed asset ratio	Fa	Fixed assets to total assets
Market competitiveness	MC	Level of competition in the manufacturing sector
Public environmental concern	PEC	Search Index for “Smog” on Baidu Search Engine

Table S2 Data corresponding to the indicators and their sources

Variables	Data Source	Original Field Name	Sources
Company Age	Wind	Current Year - Year of Incorporation	Subscribe
Number of Employees	Wind	Number of Employees	Subscribe
Fixed Assets	CSMAR	Net Fixed Assets	Subscribe
Administrative Expenses	CSMAR	Administrative Expenses	Subscribe

Revenue	CSMAR	Revenue	Subscribe
Total Liabilities	CSMAR	Total Liabilities	Subscribe
Year-End Profit	CSMAR	Operating Profit	Subscribe
Net Cash Flow from Operating Activities	CSMAR	Net Cash Flow from Operating Activities	Subscribe
Top 10 Shareholders' Ownership Ratios	CSMAR	Top 10 Shareholders' Ownership Ratios	Subscribe
Controlling Shareholder's Shareholding	CSMAR	Controlling Shareholder's Shareholding	Subscribe
Regional Sulfur Dioxide Emissions	City Statistical Yearbook	Sulfur Dioxide Emissions	Publicly available
Soot and Dust Emissions	City Statistical Yearbook	Soot and Dust Emissions	Publicly available
Digital Transformation	Juchao Information Network	Company Annual Report	Publicly available

Supplementary Information on the Development of Digital Transformation Metrics

Table S3 Keywords related to digital transformation

Table S3 Keywords related to digital transformation

Dimensions	Keywords
BD technology	Big Data, Data Mining, Text Mining, Data Visualization, Heterogeneous Data, Credit Reporting, Augmented Reality, Mixed Reality, Virtual Reality
AI technology	Artificial Intelligence, Business Intelligence, Image Recognition, Investment Decision Support Systems, Intelligent Data Analysis, Intelligent Robotics, Machine Learning, Deep Learning, Semantic Search, Biometric Technology, Facial Recognition, Speech Recognition, Identity Verification, Autonomous Driving, Natural Language Processing
blockchain technology	Blockchain, digital currency, distributed computing, differential privacy technology, smart financial contracts
cloud computing technology	Cloud computing, stream computing, graph computing, in-memory computing, multi-party secure computation, brain-inspired computing, green computing, cognitive computing, converged architecture, hundreds of millions of concurrent connections,

	exabyte-scale storage, Internet of Things, cyber-physical systems
digital technology application	Mobile Internet, Industrial Internet, Mobile Connectivity, Internet Healthcare, E-commerce, Mobile Payments, Third-Party Payments, NFC Payments, Smart Energy, B2B, B2C, C2B, C2C, O2O, NetUnion, smart wearables, smart agriculture, smart transportation, smart healthcare, smart customer service, smart home, robo-advisory, smart tourism and culture, smart environmental protection, smart grid, smart marketing, digital marketing, unmanned retail, internet finance, digital finance, Fintech, financial technology, quantitative finance, open banking

Appendix A.1 Annual Report Processing and Specific Methods for Digital Transformation：

This paper uses Python web scraping to collect and organize the annual reports of all A-share listed companies on the Shanghai Stock Exchange and the Shenzhen Stock Exchange. It then extracts all text content using the Java PDFBox library, creating a data pool for subsequent feature word screening. To identify key terms related to corporate digital transformation, this paper draws on relevant literature and consults experts to compile a list of specific keywords related to digital transformation; it further expands the feature word database by referencing important policy documents and research reports. Based on this, expressions containing negating words such as “no,” “none,” or “not” preceding the keywords were excluded. Additionally, “digital transformation” keywords referring to entities other than the company itself (including those related to shareholders, customers, suppliers, and executive profiles) were also removed. Finally, based on a data pool generated by extracting text from the annual reports of listed companies using Python, we performed searches, matches, and term frequency counts using the characteristic terms listed in the table above. We then categorized and aggregated the term frequencies related to key technology directions to form the final aggregated term frequency, thereby constructing an indicator system for

corporate digital transformation. Since this type of data exhibits a typical "right-skewed" distribution, this paper applies logarithmic transformation to it, thereby deriving an overall indicator that characterizes corporate digital transformation.

Appendix A.2 Supplementary Information on the Development of Public Environmental Concern Indicators

The core explanatory variable in this study is public environmental concern. With the development of the internet, web search data—which records user behavior through internet searches—can promptly capture market actors' attention toward specific events, reflecting their preferences and behavioral intentions; thus, environmental concern serves as a key indicator of public environmental preferences. Public environmental concern plays a role in coordinating the consistency of public environmental protection behavior; therefore, public environmental concern can be used to measure general public environmental participation. Drawing on previous research, the authors use Baidu's smog search index to characterize public environmental concern, primarily for the following reasons: as the largest Chinese-language search engine, Baidu has broad coverage and high data availability, and data from various regions of China can be analyzed based on search frequency and geographic distribution; Compared to other environmental keywords such as "environmental pollution," smog-related weather events have a relatively high level of public perception. The public can clearly gauge the severity of smog based on air visibility, and the term "smog" attracts greater public attention; therefore, netizens' interest in smog largely represents the public's overall level of environmental concern. Baidu Index is categorized by search channel into three types: Total Search Index, PC Search Index, and Mobile Search Index. The Total Search Index is calculated as the weighted sum of the PC Search Index and the Mobile Search Index.

Table S4 Benchmark Regression Results

	(1)<br>GTFP	(2)<br>GTFP
DT	0.223***	0.203***
	(0.043)	(0.034)

Age	-	-0.000
	-	(0.001)
Fin	-	0.016***
	-	(0.006)
Pro	-	0.005**
	-	(0.002)
Top10	-	-0.002
	-	(0.005)
Cash	-	0.001
	-	(0.004)
Share	-	0.000
	-	(0.010)
Ci	-	-0.037***
	-	(0.005)
Fa	-	-0.078***
	-	(0.010)
Constant	0.024***	0.073***
	(0.002)	(0.005)
Observations	13,489	13,489
Company FE	YES	YES
Year FE	YES	YES
R-squared	0.119	0.178
Observations	13,489	13,489

Note:The values in parentheses represent the standard error at the industry level， *** p<0.01, ** p<0.05, * p<0.1.

Table S5 Results of the robustness test

	(1)	(2)	(3)	(4)	(5)
	GTFP	GTFP	GTFP	GTFP	GTFP
DT	-	-	0.294***	0.202***	0.203***
	-	-	(0.050)	(0.034)	(0.026)
DTB	0.066***	-	-	-	-
	(0.014)	-	-	-	-
DTC	-	0.000*	-	-	-
	-	(0.000)	-	-	-
Age	-0.000	0.000	-0.001	-0.000	-0.000
	(0.001)	(0.002)	(0.001)	(0.001)	(0.001)
Fin	0.016***	0.013*	0.024***	0.016**	0.016***
	(0.006)	(0.007)	(0.007)	(0.006)	(0.004)
Pro	0.005**	0.005***	0.008***	0.005**	0.005**
	(0.002)	(0.001)	(0.003)	(0.002)	(0.003)
Top10	-0.002	-0.008	0.002	-0.002	-0.002
	(0.005)	(0.005)	(0.007)	(0.005)	(0.004)
Cash	0.001	0.002	0.004	0.002	0.001

	(0.004)	(0.003)	(0.005)	(0.004)	(0.007)
Share	-0.000	-0.000	0.006	-0.001	0.000
	(0.010)	(0.012)	(0.014)	(0.010)	(0.007)
Ci	-0.037***	-0.034***	-0.039***	-0.037***	-0.037***
	(0.005)	(0.004)	(0.004)	(0.005)	(0.003)
Fa	-0.079***	-0.079***	-0.085***	-0.079***	-0.078***
	(0.010)	(0.011)	(0.011)	(0.010)	(0.007)
Constant	0.072***	0.071***	0.072***	0.074***	0.073***
	(0.005)	(0.005)	(0.005)	(0.005)	(0.005)
Company FE	YES	YES	YES	YES	YES
Year FE	YES	YES	YES	YES	YES
R^2	0.1770	0.1118	0.1760	0.1770	0.1780
Observations	13,489	13,489	9,855	13,241	13,489

Note:The values in parentheses represent the standard error at the industry level.， *** p<0.01, ** p<0.05, * p<0.1.

Table S6 placebo test results

	(1)<br>Percentage of Women in Management
MDT	-0.353
	(0.327)
Age	0.009**
	(0.003)
Fin	-0.001
	(0.011)
Pro	0.011*
	(0.006)
Top10	0.015
	(0.015)
Cash	-0.005
	(0.017)
Share	-0.007
	(0.015)
Ci	-0.002
	(0.005)
Fa	0.005
	(0.014)
Observations	13,485
Number of code	2,103
R-squared	0.049

Note:The values in parentheses represent the standard error at the industry level.， *** p<0.01, ** p<0.05, * p<0.1.

Table S7 Results of the Instrumental Variables Method

	(1)<br>First Stage<br>DT	(2)<br>Second Stage<br>GTFP
DT	-	1.023**
	-	(0.375)
MDT	0.692***	-
	(0.124)	-
Age	0.002***	-0.002*
	(0.000)	(0.001)
Fin	0.004	0.012*
	(0.003)	(0.006)
Pro	-0.000	0.006**
	(0.001)	(0.002)
Top10	-0.007*	0.004
	(0.004)	(0.008)
Cash	-0.002	0.003
	(0.002)	(0.005)
Share	-0.000	0.001
	(0.003)	(0.012)
Ci	0.000	-0.036***
	(0.001)	(0.005)
Fa	-0.009***	-0.070***
	(0.002)	(0.010)
Company FE	YES	YES
Year FE	YES	YES
K–P rk LM statistic	4.834**	
K-P rk Wald F statistic	31.134	

Note:The values in parentheses represent the standard error at the industry level.， *** p<0.01, ** p<0.05, * p<0.1.

Table S8 Heckman test results

	(3)<br>First Stage<br>DT_d	(4)<br>Second Stage<br>GTFP
MDT	24.940*	-
	(15.053)	-
DT	-	0.177***
	-	(0.039)
IMR	-	0.013***
	-	(0.003)
Age	-	-0.000
	-	(0.001)

Fin	-	0.016**
	-	(0.006)
Pro	0.1091	0.007***
	(0.198)	(0.002)
Top10	-0.4701	-0.007
	(0.512)	(0.006)
Cash	-0.7924	-0.006
	(0.584)	(0.005)
Share	-0.1688	-0.001
	(0.286)	(0.011)
Ci	-0.0764	-0.038***
	(0.195)	(0.005)
Fa	-2.3176***	-0.103***
	(0.511)	(0.011)
Company FE	YES	YES
Year FE	YES	YES
Observations	13,485	13,254
R-squared/ Pseudo R2	0.6537	0.6679

Note:The values in parentheses represent the standard error at the industry level.， *** p<0.01, ** p<0.05, * p<0.1.

Table S9 Results of the moderation effect

	(1) GTFP	(2) GTFP
DT	0.134***	0.163***
	(0.028)	(0.038)
MC*DT	0.474**	
	(0.191)	
MC	-0.003	
	(0.003)	
PEC *DT		0.117***
		(0.034)
PEC		0.009***
		(0.003)
Age	-0.001	-0.001
	(0.001)	(0.001)
Fin	0.016***	0.015**
	(0.006)	(0.006)
Pro	0.005*	0.005**
	(0.003)	(0.002)
Top10	-0.002	-0.002

	(0.005)	(0.005)
Cash	0.002	0.001
	(0.004)	(0.004)
Share	0.000	0.001
	(0.010)	(0.010)
Ci	-0.037***	-0.037***
	(0.005)	(0.005)
Fa	-0.079***	-0.079***
	(0.010)	(0.010)
Constant	0.073***	0.039***
	(0.005)	(0.011)
Company FE	YES	YES
Year FE	YES	YES
Observations	13,489	13,489
R-squared	0.178	0.181

Note:The values in parentheses represent the standard error at the industry level.， *** p<0.01, ** p<0.05, * p<0.1.

Table S10 Regional and Industry Heterogeneity

	(1) Eastern Region	(2) Central Region	(3) Western Region	(4) Highly polluting industries	(5) Non-high-pollution industries
DT	0.213***	0.020	-0.002	0.219***	0.179***
	(0.038)	(0.034)	(0.066)	(0.037)	(0.059)
Age	-0.002*	-0.001	0.004	0.001	-0.002
	(0.001)	(0.001)	(0.002)	(0.002)	(0.001)
Fin	0.019***	0.018*	-0.008	0.020**	0.011
	(0.005)	(0.010)	(0.018)	(0.009)	(0.007)
Pro	0.006*	0.005**	0.003	0.005	0.008***
	(0.003)	(0.002)	(0.003)	(0.003)	(0.002)
Top10	0.001	-0.001	-0.003	-0.000	-0.008
	(0.007)	(0.007)	(0.004)	(0.007)	(0.009)
Cash	-0.002	0.000	0.021	-0.000	0.002
	(0.006)	(0.004)	(0.014)	(0.005)	(0.007)
Share	0.003	0.010	0.021*	-0.002	0.006**
	(0.013)	(0.009)	(0.011)	(0.019)	(0.002)
Ci	-0.044***	-0.020***	-0.033***	-0.045***	-0.025***
	(0.007)	(0.003)	(0.008)	(0.006)	(0.004)
Fa	-0.097***	-0.037***	-0.048***	-0.088***	-0.072***
	(0.014)	(0.010)	(0.016)	(0.013)	(0.011)
Constant	0.084***	0.044***	0.058***	0.083***	0.058***
	(0.005)	(0.008)	(0.013)	(0.007)	(0.003)
Company FE	YES	YES	YES	YES	YES

Year FE	YES	YES	YES	YES	YES
Observations	9,135	2,551	1,803	7,819	5,670
R-squared	0.183	0.357	0.248	0.162	0.241
Number of code	9,135	2,551	1,803	7,189	5,670

Note:The values in parentheses represent the standard error at the industry level.， *** p<0.01, ** p<0.05, * p<0.1.

Table S11 Firm-level heterogeneity

	(1)<br>Early stages	(2)<br>Later stages	(3)<br>Large-scale Enterprises	(4)<br>Small-scale Enterprises
DT	0.028	0.105***	0.247***	0.163***
	(0.027)	(0.030)	(0.049)	(0.043)
Age	-0.002**	0.001	-0.002	0.003*
	(0.001)	(0.001)	(0.001)	(0.002)
Fin	0.001	0.007	0.024**	0.009
	(0.007)	(0.007)	(0.009)	(0.009)
Pro	0.008	-0.001	0.007	0.003
	(0.006)	(0.003)	(0.004)	(0.003)
Top10	-0.009*	-0.004	-0.002	0.004
	(0.004)	(0.007)	(0.004)	(0.010)
Cash	-0.007	-0.007	-0.009	-0.002
	(0.011)	(0.005)	(0.010)	(0.007)
Share	-0.009	0.015	0.003	-0.001
	(0.006)	(0.011)	(0.007)	(0.017)
Ci	-0.023***	-0.042***	-0.031***	-0.041***
	(0.005)	(0.007)	(0.004)	(0.007)
Fa	-0.046***	-0.089***	-0.060***	-0.098***
	(0.012)	(0.009)	(0.012)	(0.016)
Constant	0.064***	0.090***	0.064***	0.082***
	(0.008)	(0.006)	(0.008)	(0.008)
Company FE	YES	YES	YES	YES
Year FE	YES	YES	YES	YES
Observations	4,139	9,350	6,745	6,744
R-squared	0.095	0.130	0.202	0.151
Observations	4,139	9,350	6,745	6,744

Note:The values in parentheses represent the standard error at the industry level.， *** p<0.01, ** p<0.05, * p<0.1.

Discussion on Effect Size and Empirical Positioning

To further situate our findings within the existing literature, it is useful to compare the magnitude of the estimated effect of digital transformation on green total factor

productivity (GTFP) with related studies on digital transformation and productivity outcomes. Prior studies on digital transformation and firm productivity generally report positive but moderate effects, reflecting improvements in resource allocation and operational efficiency. In the context of environmentally constrained productivity, existing evidence on digitalization and green productivity also suggests a positive association, although the estimated magnitudes vary depending on measurement approaches and environmental indicators.

Against this backdrop, the estimated coefficients in this study indicate a relatively robust and economically meaningful effect of digital transformation on GTFP in the manufacturing sector. This suggests that digital transformation not only enhances conventional productivity but also contributes to productivity improvements under environmental constraints, particularly those associated with air pollutant emissions such as SO_2 and soot.

Compared with studies focusing solely on conventional total factor productivity, the effect size observed in this study is generally more sensitive to institutional and market conditions, as evidenced by the significant moderating roles of market competition and public environmental awareness. This heterogeneity highlights that the productivity-enhancing effects of digital transformation are contingent on external environmental pressures, which may strengthen firms' incentives to translate digital capabilities into efficiency gains under environmental constraints.

Data and Code Availability

This study is committed to upholding high standards of academic transparency and research reproducibility. The core Stata replication scripts (.do code files), detailed variable construction specifications, the complete digital transformation keyword dictionary, and the cleaned, anonymized enterprise-level panel dataset used in this study have all been publicly uploaded to our replication repository. Readers can use these codes and data to fully reproduce all benchmark regression, heterogeneity analysis, and mechanism test results presented in this study.

It should be noted that, due to copyright and privacy restrictions imposed by third-party data providers, access to some of the original microdata sets (such as the China Industrial Enterprises Green Development Database) requires readers to have a subscription or access rights through specific institutions. To enhance transparency, we have provided a detailed classification and description of the acquisition channels and subscription requirements for each data source in Appendix Table 2.
